# Extraction and process optimization of *bael* fruit pectin

**DOI:** 10.1002/fsn3.761

**Published:** 2018-08-23

**Authors:** Bunty Maskey, Drishya Dhakal, Mahalaxmi Pradhananga, Nabindra Kumar Shrestha

**Affiliations:** ^1^ Department of Food Technology Central Campus of Technology Tribhuvan University Dharan Nepal; ^2^ Sunsari Technical College Tribhuvan University Dharan Nepal; ^3^ Central Campus of Technology Tribhuvan University Dharan Nepal

**Keywords:** *bael*, gels, methoxylated polygalacturonic acid, pectin, polysaccharides

## Abstract

Pectin was extracted at temperature of 100 and 120°C with two different pH conditions each, pH 1.5 and pH 2 along with different extraction ratios, ER1:1, 1:1.5 and 1:2 respectively. Data obtained were analyzed statistically using ANOVA (no blocking) at 5% level of significance, which showed that there was significant difference (*p* < 0.05) between the pectin extracted at 100°C, pH 1.5 and extraction ratios of 1:1, 1:1.5, and 1:2, respectively, whereas there was not any significant difference between extraction at 120°C, pH 1.5 and extraction ratios of 1:1, 1:1.5, and 1:2 respectively. Also, there was no any significant difference between pectin extracted at 100°C, pH 2, extraction ratios 1:1, 1:1.5, and 1:2 and also at 120°C, pH 2, 1:1, 1:1.5, and 1:2 respectively. Best yield of pectin was found at temperature 120°C, pH 1.5 and ER 1:1.5.

## 1 INTRODUCTION

Pectin (derived from Greek meaning “congealed and curdled”) is a structural hetero polysaccharide containing in the cell of terrestrial plant. It was first isolated and described by Heneri Bracannot in 1825. Pectin is a purified carbohydrate product obtained from the inner portion of the rind/peels of citrus fruits. It consists chiefly of partially methoxylated polygalacturonic acid. Pectin is capable of forming gels with sugar and acid under suitable conditions. It is formed almost universally in plant cell of all species suitable for use in the production of sugar jellies and industrial production of apple pomace, citrus peels, and sugar beet chips. Pectin extracted from various materials can be different in molecular structure (i.e., molecular weight, degree of esterification, and acetyl content) and therefore possesses different functional properties. Typically, a whole mature fruit contains 3%–7% pectin substances on a dry weight basis and 0.1%–1.1% on a fresh weight basis. The relatively high pectin and low caloric content of citrus fruits make them a good source of soluble dietary fiber (Joslyn, 1980).

A valuable by product that can be obtained from fruit wastes is pectin. Pectin designates those water soluble pectinic acid (colloidal polygalacturonic acids) of varying methyl ester content and degree of neutralization which is capable of forming gels with sugar and acids under suitable conditions. The suitability of pectin for different purposes is determined by their character via, anhydrouronic acid content (AUA) methoxyl content, degree of esterification, and acetyl values. Pectin, the gelatinizing agent is natural to fruits and it also occur in many other vegetable products but the amount and quality of pectin depend upon the type of fruits and vegetables maturity and method adopted for the extraction of pectin (GITCO, 1999).

The *bael* fruit is known for its medicinal properties and is one of the most nutritious fruits. It contains 61.5 g of water, 1.8 g of protein, 1.7 g of minerals, 31.8 g of carbohydrates, and 1.19 mg of riboflavin/100 g edible portion. Its food value is 88 calories/100 gm. Thus, it is richer than most of the reputed fruits like apple, guava, and mango which have a calorific value of only 64, 59 and 36 respectively. It may be noted that no other fruits have such a high content of riboflavin. *Bael* fruit can be stored for 10–15 days at normal temperature, whereas fruit harvested at ripe stage can be stored for a week. After extraction of *bael* pulp used for the preparation of various fruit products viz. preserve, candy, jam, RTS, nectar squash/leather/slab, powder etc., which can be commercially exploited (ITDG, 2002).

This research is about pectin extraction from *bael* fruit which has anti‐microbial activity. It gives clear edge over commercial citrus pectin for exploitation in food and pharmaceutical. *Bael* fruit is an under‐utilized fruit. Though abundantly found, its products are limited to home scale only. The commercial value is very low. *Bael* has very strong taste, its consumption as fruit is not generally preferred. It is highly nutritious in terms of vitamin and dietary fiber. The processing of bael products are in a primitive stage. Hence, this research may contribute in proper utilization of bael fruit for production of high quality pectin which can increase its demand and therefore generate income for farmers and distributers.

## 2 MATERIALS AND METHODOLOGY

*Bael*s were purchased from local retailers for use in experiments. The experiment required a larger amount of pulp so the individual trials were conducted using different *bael*s of the same variety and similar maturity level. The *bael*s were washed carefully with tap water to remove dirt soil from surface and broken on hard surface. The pulp was extracted by spoon. After pulp extraction, it was dried at 60°C for 24 hr in a cabinet drier followed by grinding into powder by using a blender. The powder was sieved using sieve and packed in low density polyethylene bag (thickness 75 μm). The obtained powder was sealed and stored at 6–10°C for further study.

### 2.1 Pectin extraction

300 ml of distilled water was measured into a 1,000 ml conical flask and desired temperature was maintained using hot plate or the shaking water bath. A weighed portion of prepared pulp powder (50 gm) was added to the water and measured amount of acid was added to the pulp–water mixture until the desired pH was obtained. The mixture was agitated at a constant temperature until the desired extraction time (60 min) had elapsed. The pHs and temperatures were recorded and the mixture was allowed to cool in an ice water bath until it reached 55°C, the mixture was then centrifuged at 257 g for 10 min.

The filtrate was vacuum filtered using Whatman filter paper and the centrifugation and filtration steps were repeated. The filtered solutions were combined and approximately the volume of alcohol and filtered solution was maintained for different extraction ratios in different conical flasks, precipitation was allowed overnight. The next day, pectin was separated from the alcohol solution using a double layer of muslin cloth and washed three times with 70% alcohol and once with undiluted alcohol to remove any impurities. The pectin was dried in hot air oven until all moisture was removed. Samples were cooled and weighed. The samples were then stored in small plastic sample bags for further study (Pistoia, 1976).

### 2.2 Characterization of extracted pectin

The dried pectin obtained was subjected to the following tests for characterization.

1. *Color*: Visual observation was done for observing color of extracted pectin from the process given by Karki and Thapaliya (2004).
2. *Equivalent weight*: Equivalent weight was determined by process given in Ranganna, 1995. Equivalent wt. is used for calculating the AUA and the degree of esterification.
3. *Methoxyl content (MeO)*: Determination of MeO was done by method given in Ranganna, 1995.
4. *Moisture content*: Moisture content of pectin was determined by hot air oven method given by Ranganna, 2011.
5. *Total* AUA: Pectin which is a partly esterified polygalacturonide, contains 10% or more of organic material composed of arabinose, galactose and perhaps sugars. Estimation of AUA is essential to determine the purity and degree of esterification, and to evaluate the physical properties. Total AUA of pectin was obtained by the following formula (Mohamed & Hasan, 1995).

$$\%\;\text{of}\;\text{AUA}=\frac{176\times 0.1z\times 100}{W\times 1000}+\frac{176\times 0.1y\times 100}{W\times 1000}$$

where, *z* = ml (titer) of NaOH from equivalent weight determination, *y* = ml (titer) of NaOH from methoxyl content determination, W = weight of sample.

- *Degree of esterification (DE)*: The DE of pectin was measured on the basis methoxyl and AUA content (Owens et al., 1952) and calculated by following formula.

$$\%\text{DE}=\frac{176\times \%\text{MeO}}{31\times \%\text{AUA}\times 100}$$

- *Ash content*: Ash content was determined by dry ashing method given by Ranganna, 2011.
- *Pectin grades*: Pectin grade was determined by grading method given in Ranganna, 2011.
- *Pectin yield*: Yield of pectin was determined according to process given in Ranganna, 2011.

The data obtained from the yield of pectin in different conditions were analyzed by two ways ANOVA (no blockings) at 5% level of significance. The yields were analyzed by using LSD method (GenStat 5 Release 7.1 software programme developed by Lawes Agricultural Trust, Rothamsted Experimental Station, 1985). Means of the data were separated whether they are significant or not by using Fisher's LSD (least significant difference) method at 5% level of significance.

## 3 RESULTS AND DISCUSSION

The present study was carried out to optimize the process of pectin extraction from Bael fruit. The pulp of bael was used for extraction. Extraction was carried out by alcohol precipitation method at given temperature and pH. Extraction process was varied in temperature, pH and ethanol extraction ratio i.e., temperature 100 and 120°C, pH 1.5 and 2, ER 1:1, 1:1.5, and 1:2 respectively. Finally, the condition of temperature, pH and ER with highest pectin yield was taken as the best one. During the work, the chemical composition of bael pulp, the chemical composition of extracted pectin and yield of pectin at each varying conditions were studied.

### 3.1 Analysis of fresh bael pulp

The pulp of bael was analyzed for moisture, carotene, TSS, vitamin C, color, and taste and the following results were obtained as shown in Table 1.

**Table 1:** Analysis of *bael* pulp

Parameter	Result
Moisture content (%)	82.9 ± 0.29
Vitamin‐C	11.7 ± 0.06 mg/100 gm
TSS (°Bx)	8.5 ± 0.1
Carotene	12 ± 0.36 mg/100 gm
Color	Creamy yellow
Taste	Sweet

### 3.2 Extraction yield at different sets of combination of temperature, pH, and extraction ratios

#### 3.2.1 Extraction yield at 100°C, pH 1.5, and ER 1:1

Three samples were made namely A_11_, A_12_, and A_13_. Pectin was extracted at 100°C, pH 1.5 and ER 1:1 for each of the samples. The mean of yields is considered as A_1_ shown in Table 2.

**Table 2:** Yield at 100°C, pH 1.5, and ER 1:1

Sample	Product (gm)	(%) Yield
A_11_	1.32	2.64
A_12_	1.09	2.18
A_13_	1.21	2.42

#### 3.2.2 Extraction yield at 100°C, pH 1.5, and ER 1:1.5

Also at 100°C, three samples were made namely B_11_, B_12_, and B_13_. For each of the sample extraction was done at 100°C, pH 1.5, and ER 1:1.5. The mean of yields is considered as B_1_ shown in Table 3.

**Table 3:** Yield at 100°C, pH 1.5, and ER 1:1.5

Sample	Product (gm)	(%) Yield
B_11_	2.32	4.64
B_12_	2.08	4.16
B_13_	2.21	4.42

#### 3.2.3 Extraction yield at 100°C, pH 1.5, and ER 1:2

Three samples were prepared namely C_11_, C_12_, and C_13_. Extraction was done for each of the samples. The mean of yields is considered as C_1_ shown in Table 4.

**Table 4:** Yield at 100°C, pH 1.5, and ER 1:2

Sample	Product (gm)	(%) Yield
C_11_	1.46	2.92
C_12_	1.36	2.72
C_13_	1.42	2.82

#### 3.2.4 Extraction yield at 100°C, pH 2, and ER 1:1

Three samples were prepared namely A_21_, A_22_, and A_23_. Extraction was done for each of samples. The mean of yields is considered as A_2_ shown in Table 5.

**Table 5:** Yield at 100°C, pH 2, and ER 1:1

Sample	Product (gm)	(%) Yield
A_21_	1.10	2.2
A_22_	1.27	2.54
A_23_	1.19	2.38

#### 3.2.5 Extraction yield at 100°C, pH 2, and ER 1:1.5

Three samples were prepared namely B_21_, B_22_, and B_23_. Extraction was done for each of samples. The mean of yields is considered as B_2_ shown in Table 6.

**Table 6:** Yield at 100°C, pH 2, and ER 1:1.5

Sample	Product (gm)	(%) Yield
B_21_	2.23	4.46
B_22_	2.30	4.6
B_23_	2.18	4.36

#### 3.2.6 Extraction yield at 100°C, pH 2, and ER 1:2

Three samples were prepared namely C_21_, C_22_, and C_23_. Pectin extraction was done for each of the samples. The mean of yields is considered as C_2_ shown in Table 7.

**Table 7:** Yield at 100°C, pH 2, and ER 1:2

Sample	Product (gm)	(%) Yield
C_21_	1.10	2.2
C_22_	1.17	2.34
C_23_	1.20	2.4

#### 3.2.7 Extraction yield at 120°C, pH 1.5, and ER 1:1

Three samples were prepared namely A_31_, A_32_, and A_33_. Pectin extraction was done for each of the samples. The mean of yields is considered as A_3_ shown in Table 8.

**Table 8:** Yield at 120°C, pH 1.5, and ER 1:1

Sample	Product (gm)	(%) Yield
A_31_	3.36	6.72
A_32_	3.42	6.84
A_33_	3.36	6.72

#### 3.2.8 Extraction yield at 120°C, pH 1.5, and ER 1:1.5

Three samples were prepared namely B_31_, B_32_, and B_33_. Pectin extraction was done for each of the samples. The mean of yields is considered as B_3_ shown in Table 9.

**Table 9:** Yield at 100°C, pH 2, and ER 1:1

Sample	Product (gm)	(%) Yield
B_31_	7.88	15.76
B_32_	8.03	16.06
B_33_	8.35	16.7

#### 3.2.9 Extraction yield at 120°C, pH 1.5, and ER 1:2

Three samples were prepared namely C_31_, C_32_, and C_33_. Pectin extraction was done for each of the samples. The mean of yields is considered as C_3_ shown in Table 10.

**Table 10:** Yield at 100°C, pH 2, and ER 1:1

Sample	Product (gm)	(%) Yield
C_31_	2.52	5.04
C_32_	2.68	5.36
C_33_	2.79	5.58

#### 3.2.10 Extraction yield at 120°C, pH 2, and ER 1:1

Three samples were prepared namely A_41_, A_42_, and A_43_. Pectin extraction was done for each of the samples. The mean of yields is considered as A_4_ shown in Table 11.

**Table 11:** Yield at 100°C, pH 2, and ER 1:1

Sample	Product (gm)	(%) Yield
A_41_	4.46	8.92
A_42_	5.72	11.44
A_43_	5.80	11.6

#### 3.2.11 Extraction yield at 120°C, pH 2, and ER 1:1.5

Three samples were prepared namely B_41_, B_42_, and B_43_. Pectin extraction was done for each of the samples. The mean of yields is considered as B_4_ shown in Table 12.

**Table 12:** Yield at 100°C, pH 2, and ER 1:1

Sample	Product (gm)	(%) Yield
B_41_	2.73	5.46
B_42_	3.81	7.62
B_43_	2.02	4.04

#### 3.2.12 Extraction yield at 120°C, pH 2, and ER 1:2

Three samples were prepared namely C_41_, C_42_, and C_43_. Pectin extraction was done for each of the samples. The mean of yields is considered as C_4_ shown in Table 13.

**Table 13:** Yield at 100°C, pH 2, and ER 1:1

Sample	Product (gm)	(%) Yield
C_41_	2.71	5.42
C_42_	1.52	3.04
C_43_	2.32	4.64

From above tables, pectin extraction and yield at 120°C, pH1.5 and ER 1:1.5, sample B_3_ was highest i.e., 16.7% which was similar to amarelle peel pectin (13% to 17%) and mango peel pectin (4.6% to 18.5%) (Koubala et al., 2008). However, the yield of *bael* pulp pectin was higher than that as reported by Yapo (2009) for passion fruit (7.5%) and lower than golden apple (22%) (Rha et al., 2011).

### 3.3 Chemical analysis

The moisture content of extracted pectin was found 16.66%. This observation was comparable to that found by Ismail et al. (2012) who reported moisture content of dragon fruit pectin varied from 11.13% to 17.33%. The ash content was found to be 1.24%. The ash content increases as the pectin yield decreases, indicating that the sugar content and others constituent increases significantly due to ripening of the fruit. Low ash content i.e., below 10% and maximum limit of ash content 10% are one of the good criteria for gel formation (Ismail et al., 2012). Therefore, the ash content found in this experiment indicates the purity of the pectin. The pectin produced in this study can be categorized as low methoxyl pectin (LMP) 13.39%. Also, it has a %DE lower than 50% so it can be considered as rapid set pectin. The AUA was found to be 73.21 which showed that the pectin was pure. Also, the pectin isolated was of 150 grade with whitish yellow color and disagreeable odor. The chemical analysis of sample B_3_ (extracted at 1200C, pH 1.5 and ER 1:1.5) as shown in Table 14.

**Table 14:** Chemical analysis of sample B_3_ (extracted at 1,200°C, pH 1.5, and ER 1:1.5)

Parameters	Yield (%)
Wet basis	16.7 ± 0.3
Dry basis	20.02 ± 0.3
Moisture content	16.66 ± 0.64
Equivalent weight	1,488.09 ± 0.29
Methoxyl content	13.39 ± 0.4
Ash content	1.24 ± 0.036
Anhydrous acid content	73.21 ± 0.07
Degree of esterification	47.2 ± 1.02
Pectin grade	150
Color	Whitish yellow
Odor	Dis‐agreeable

### 3.4 Variation in ER at temperature 100°C and pH 1.5

**Figure 1:**
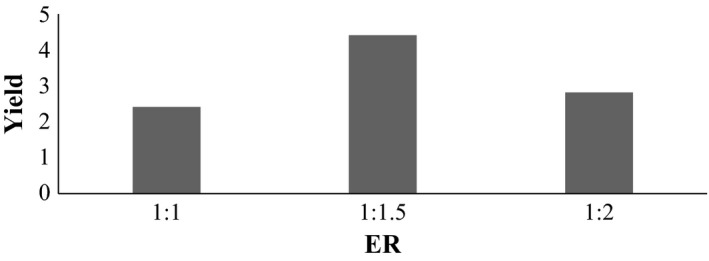
Effect on extraction yield at temperature 100°C and pH 1.5

Here, A_1_, B_1_, and C_1_ are same set of samples but subjected to different ER i.e., 1:1, 1:1.5, and 1:2, respectively, shown in Figure 1, between the samples extracted at 100°C and pH 1.5, the % yield is significantly different from each other. As LSD was found to be 0.1775, it shows that sample extracted at ER 1:1.5 is significantly different from sample extracted at 1:1 and 1:2. From figure above, it shows that mean score of sample extracted at ER 1:1, 1:1.5, and 1:2 are 2.413%, 4.407%, and 2.820% respectively. So, sample B_1_ (extracted at ER 1:1.5) was found to be best in terms of % yield.

### 3.5 Variation in ER at temperature 100°C and pH 2

**Figure 2:**
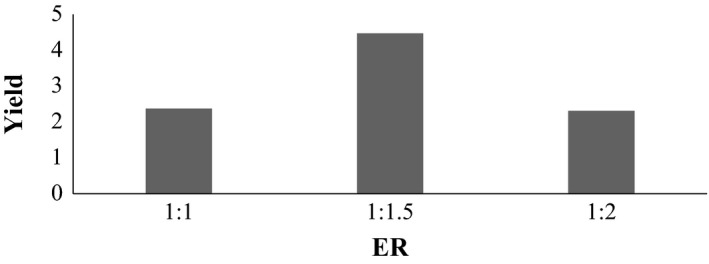
Effect on extraction yield at temperature 100°C and pH 2

Also here, let A_2_, B_2_, and C_2_ be samples extracted at ER 1:1, 1:1.5, and 1:2, respectively, as shown in Figure 2. The sample extracted at 100°C and pH 2, all the samples were identical. There was significant difference between the samples. Thus, % yield was taken as the basis for best sample selection. So, sample B_2_ extracted at ER 1:1.5 was taken as best sample. From figure above, it shows that mean score of sample A_2_, B_2_, and C_2_ are 2.373%, 4.473%, and 2.313% respectively. So, sample B_2_ has the highest % yield then sample A_2_ and C_2_.

### 3.6 Variation in ER at temperature 120°C and pH 1.5

**Figure 3:**
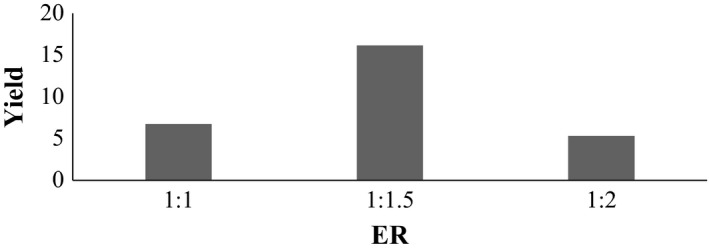
Effect on extraction yield at temperature 120°C and pH 1.5

Samples A_3_, B_3_, and C_3_ are pectin extracted at ER of 1:1, 1:1.5, and 1:2 respectively shown in Figure 3. Among the sample extracted at 120°C, pH 1.5, the % yield was similar to each other. There was significant difference between the samples. From the figure it shows that mean score of sample A_3_, B_3_, and C_3_ was 6.760%, 16.173%, and 5.327% respectively. So, sample B_3_ was found to be best in terms of % yield.

### 3.7 Variation in ER at temperature 120°C and pH 2

**Figure 4:**
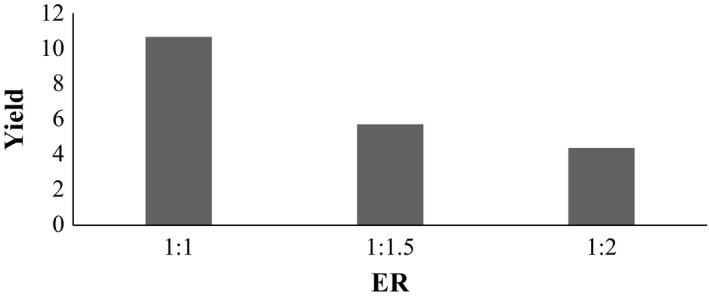
Effect on extraction yield at temperature 120°C and pH 2

Samples A_4_, B_4_, and C_4_ are samples at ER of 1:1, 1:1.5, and 1:2 respectively in above Figure 4. The sample extracted at 120°C and pH 2, the % yield are similar to each other. It shows that sample A_4_ extracted at (ER 1:1) was found to be best in %yield. From figure it shows that the mean score of sample A_4_, B_4_, and C_4_ was 10.65%, 5.71%, and 4.37% respectively. So, sample A_4_ was found to be best in terms of % yield.

### 3.8 Comparison between the samples

Comparison between the samples B_1_ (extracted at pH 1.5 and ER 1:1.5) and B_2_ (extracted at pH 2 and ER 1:1.5) was done statistically. The yield was found to be similar. Between samples B_3_ (extracted at pH 1.5 and ER 1:1.5) and A_4_ (extracted at pH 2 and ER 1:1) yield was found to be similar. Thus % yield was taken as the best sample selection. So, sample B_3_ (extracted at pH 1.5 and ER 1:1.5 and temperature 1,200°C) was taken as the best among the other samples.

## 4 CONCLUSIONS

The best condition for pectin extraction was at pH 1.5, ER 1:1.5 and temperature 120°C. The yield was found to be 16.7%. The %AUA was mostly above 65%, which indicates that the pectin was pure. So, it might be used as functional food ingredient domestically and industrially. Bael pulp gave higher yield with low methoxyl content, high AUA and greater DE. So, it can be utilized as rich source of pectin and can be commercially exploited. From the results of this study, bael pulp gave significant amount of pectin. So, it can be used and utilized to make high quality pectin at lower cost.

## CONFLICT OF INTEREST

None declared.
